# Interactive effects of FR and UV-A on growth and phytochemical content of basil and parsley grown in an indoor vertical farm

**DOI:** 10.3389/fpls.2026.1843306

**Published:** 2026-07-22

**Authors:** Md Obyedul Kalam Azad, Most Tahera Naznin

**Affiliations:** Department of Agriculture, Veterinary and Rangeland Sciences, University of Nevada, Reno (UNR), UNR Extension and Agricultural Experimental Station, Las Vegas, NV, United States

**Keywords:** antioxidant capacity, baby herbs, pigment content, plant growth, supplemental light

## Abstract

Light spectrum and intensity are key environmental factors regulating plant growth, photosynthetic performance, and secondary metabolite accumulation. In this study, we aimed to evaluate the effects of FR light and investigate how adding UV-A to the FR treatment influenced the growth and phytochemical content of basil and parsley under different light intensities. Light spectrum and intensity are key environmental factors regulating plant growth, photosynthetic performance, and secondary metabolite accumulation. Light treatments were arranged with three spectra and intensities (25, 50, and 100 µmol m^−2^ s^−1^) as T1 (RBW-25), T2 (RBW+FR-25), T3 (RBW+FR+UV-A-25), T4 (RBW-50), T5 (RBW+FR-50), T6 (RBW+FR+UV-A-50), T7 (RBW-100), T8 (RBW+FR-100), and T9 (RBW+FR+UV-A). In basil, T8 significantly increased plant height, leaf number, and biomass accumulation compared with the corresponding RBW treatment (T7). Leaf area was greatest under T3 and T6, whereas T4 promoted root elongation. In parsley, T2 increased plant height, while T5 enhanced leaf area and fresh biomass accumulation. Total phenolic content (TPC) and total flavonoid content (TFC) in basil were highest under T5 and T8, whereas T3, T6, and T9 enhanced these metabolites in parsley. Regarding pigment accumulation, T8 markedly increased chlorophyll *a*, chlorophyll *b*, and carotenoid in basil, while in parsley, T9 produced the highest chlorophyll accumulation, and T8 resulted in the greatest carotenoid content. In basil, *F*_v_/*F*_m_ increased from 0.73 in T1 to 0.81 in T2, while PI increased from 0.98 to 3.00.Stomatal conductance was highest under T6 (327 mmol m^−2^ s^−1^). In parsley, *F*_v_/*F*_m_ remained relatively stable among treatments, whereas PI was highest under T4 (1.68), and g_s_ increased substantially under T7 (259 mmol m^−2^ s^−1^). Anthocyanin accumulation in basil was greatest under T9, with values approximately 46% higher than the corresponding RBW. Antioxidant capacity determined by DPPH and FRAP assays was consistently enhanced under T2 and T8 in basil, whereas T2 and T6 produced the highest antioxidant activity in parsley. Principal component analysis (PCA) explained 62.8% of the total variance, with PC1 and PC2 accounting for 41.3% and 21.5%, respectively, and revealed distinct treatment-associated separation patterns. Basil was primarily associated with chlorophylls, carotenoids, stomatal conductance, and amino acid accumulation, whereas parsley was more closely associated with nitrate content, phenolics, flavonoids, and antioxidant activity. Overall, FR supplementation primarily enhanced growth and photosynthetic performance, whereas the combined application of FR and UV-A shifted plant metabolism toward antioxidant activity and secondary metabolite accumulation. These responses indicate that UV-A may either complement or antagonize FR effects depending on irradiance level, physiological trait, and crop species under controlled-environment conditions.

## Introduction

Basil (*Ocimum basilicum* L.) and parsley (*Petroselinum crispum* L.) are widely cultivated culinary herbs valued for their aromatic properties and health-promoting phytochemicals, including phenolics, flavonoids, and antioxidant compounds. Basil is generally characterized by greater photosynthetic adaptability and stronger responsiveness in secondary metabolite biosynthesis, whereas parsley typically exhibits more stable growth patterns and limited pigment adjustment under controlled environmental conditions (Shahrajabian et al., 2020; Bahramsoltani et al., 2024). These physiological differences suggest that basil and parsley may respond differently to varying spectral light combinations. Red and blue spectra are the primary drivers of photochemical processes, directly influencing chlorophyll absorption, photosystem excitation, and carbon assimilation. Blue light regulates stomatal conductance, chloroplast development, and growth responses (Hernández and Kubota, 2016; Hogewoning et al., 2010).

Beyond the red and blue spectra, far-red function as regulatory signals that modulate plant architecture, resource allocation, and secondary metabolism without substantially increasing photosynthetic photon flux density (PPFD) (Laužikė et al., 2023). Far-red light plays a central role in shade-avoidance responses by mediating phytochrome perception of the red:far-red ratio, thereby influencing downstream hormonal signaling and gene expression. Far red has been shown to promote stem elongation, leaf expansion, canopy light interception, and biomass accumulation (Franklin and Whitelam, 2005; Zhen and Bugbee, 2020). In controlled-environment agriculture (CEA), far-red light enhances photosynthetic performance indirectly by improving leaf morphology, reducing self-shading, and increasing the balance of photosystem excitation between PSI and PSII, thereby improving overall light-use efficiency (Meng et al., 2024; Carotti et al., 2024). In addition, UV-A activates cryptochrome-associated signaling pathways, inducing the biosynthesis of phenolic compounds, flavonoids, anthocyanins, and other antioxidant metabolites (Taulavuori et al., 2018). Under CEA conditions, UV-A has been reported to enhance nutritional quality and antioxidant capacity in leafy vegetables and herbs (Samuolienė et al., 2012). Moreover, UV-A radiation is less harmful to plant tissues due to its longer wavelength and lower photon energy, which results in reduced cellular damage (Choi et al., 2022).

Despite extensive research on FR and UV-A supplementation, most studies have been conducted at relatively high PPFD (>150 to 350 µmol m^−2^ s^−1^), leaving the combined effects under low PPFD (25–100 µmol m^−2^ s^−1^) poorly understood. This gap is particularly relevant for energy-efficient plant factory and vertical farming systems, where crops are frequently cultivated under reduced irradiance to minimize electricity consumption and improve production sustainability. Under such low-light conditions, plants may rely more strongly on spectral signaling than photon quantity, thereby altering growth, photochemical performance, and metabolic partitioning in response to light-quality interactions (Butturini and Marcelis, 2020; Pennisi et al., 2019). Although several studies have examined supplemental lighting strategies, most have focused on the individual effects of FR radiation (Kelly and Runkle, 2024) or UV-A radiation (Laužikė et al., 2023; Kim and Eom, 2025) under optimal growth conditions rather than on their interactive effects at such a lower irradiance. We therefore hypothesized that, under low PPFD conditions, supplemental FR would enhance plant growth and photochemical performance, while adding UV-A to the FR treatment would further modify secondary metabolite accumulation compared with FR alone.

## Materials and methods

### Plant materials and growing conditions

Basil (*Ocimum basilicum* L. cv. MIA Prosper active DMR) and parsley (*Petroselinum crispum* L cv. Katinka) seeds (Johnny seeds.com, USA) were germinated on moistened 1.2-inch rockwool cubes (Grodan, Roermond, Netherlands). After emergence (three weeks after sowing), uniform seedlings were selected and transferred to a deep-flow hydroponic system (a 10-L-capacity plastic container) using a commercial nutrient solution (Humboldt’s Secret Supplies, California, USA). The nutrient solution was applied at 1.6 ml/L of water, according to the manufacturer’s dosage guidelines. Both plant species were grown in an indoor plant factory at 23 ± 2°C, 35–40% relative humidity, and a 16 h light/8 h dark photoperiod. The hydroponic nutrient solution was maintained at an EC of 1.5–2.0 dS m^−1^ and pH 6.0 to 6.5, adjusted as needed using pH down phosphoric acid (General hydroponics, Rosa, CA, USA). The solution was aerated continuously and replaced every 2 weeks to prevent nutrient/salt deposition. The experiment was repeated twice, and plants were randomly assigned to each light treatment to minimize positional effects within the indoor growth system. For plant growth measurements, twelve plants per treatment were harvested and analyzed for morphological and biochemical parameters, while six plants/treatment/replication were used for chlorophyll fluorescence, stomatal conductance, and biochemical measurements. Six plants from each treatment/replication were dried together and made into a powder, generating three technical replicates (n=3) for statistical analysis.

### Artificial LED light treatments

Five artificial LED light spectra, such as red (R) 660 nm, blue (B) 430 nm, far red (FR) 730 nm, UVA, 320 nm and white (W) cool white (correlated color temperature CCT 6500K) were used (LED panel grow light.com, China). Three spectral combinations were used based on the ratio of spectral intensities of total PPFD as follows: RBW = 1:1:3; RBW: FR = 1:1:3:0.5; and RBW: FR: UVA = 1:1:3:0.5:0.5. Each light combination had three different light intensities 100, 50 and 25 µmol m^−2^ s^−1^ as demonstrated in **Table 1**. Total PPFD was measured at the canopy level using a calibrated quantum sensor (LI-250A, LI-COR Biosciences, USA). Adjustments were made by modifying the panel height to compensate for changes in plant growth across all treatments.

**Table 1:** Artificial LED light treatment combinations.

Spectra combinations	Intensity (± 10 µmol m^−2^ s^−1^)	Treatments
RBW	25	T1
RBW+FR	25	T2
RBW+FR+UVA	25	T3
RBW	50	T4
RBW+FR	50	T5
RBW+FR+UVA	50	T6
RBW	100	T7
RBW+FR	100	T8
RBW+FR+UVA	100	T9

Because an RBW + UV-A treatment was not included, the observed responses will be interpreted as the combined effects of FR and UV-A rather than conclusive evidence of synergistic or antagonistic interactions.

### Analysis of maximum quantum yield and stomatal conductance

Maximum quantum yield (F_v_/F_m_), performance index (PI) and stomatal conductance (g_s_) were measured on 30 days post-transplantation of basil and parsley. The F_v_/F_m_ was measured using a chlorophyll fluorometer after 30 min dark adaptation, along with PI using a portable chlorophyll fluorimeter (Hansatech Pocket PEA Chlorophyll Fluorimeter). The gs was measured using SC-1 Leaf Porometer (Meter Group Inc., Pullman, WA, USA). In each treatment, six plants per treatment were used for data collection.

### Plant morphological characteristics measurements

After thirty days post-transplantation, twelve plants from each treatment/study (24 plants total) were harvested for morphological and biochemical analyses. The following parameters were recorded including plant height (cm): measured from the base to the apex using ruler; leaf number: total fully expanded leaves per plant; leaf area (cm²): measured using a leaf area meter (LI-3100, LI-COR, USA); root length (cm): measured using ruler; fresh weight (FW) and dry weight (DW) using an electric balance. Dry weight was determined after oven-drying samples at 65°C for 72 h.

### Plant polyphenol and flavonoid analysis

#### Sample preparation

For the determination of total polyphenol content (TPC), total flavonoid content (TFC), total free amino acids, nitrate concentration, and antioxidant capacity, twelve plants from each replication were collected for each plant species. Antioxidant capacity was evaluated using the 2,2-diphenyl-1-picrylhydrazyl (DPPH) radical-scavenging assay to assess free-radical scavenging activity and the ferric reducing antioxidant power (FRAP) assay to determine the reducing capacity of the plant extracts. Two sets of plants were dried and ground into a fine powder, then kept in an airtight ziplock bag. Briefly, 0.5 g of dried sample was mixed with 30 mL of 80% methanol in a 50 mL falcon plastic tube. The mixture was shaken at 300 rpm overnight (12 to16 hr) using an electric shaker (Thermo Fisher Scientific, Bohemia, NY, USA). The extract was then filtered through Whatman No. 1 filter paper (0.20 mm, Whatman, *5B* Cytiva, Marlborough, MA, USA), and the eluate was stored at 4°C until further analysis.

#### Estimation of total phenolic content

The total phenolic content (TPC) of basil and parsley was quantified according to Folin–Ciocalteu method (Singleton and Rossi, 1965). Briefly, 1 mL of the extract was mixed with 0.2 mL of 1 N Folin–Ciocalteu reagent in a test tube. The mixture was then increased by adding 1.8 mL of deionized water, vortexed, and allowed to react for 3 min. Subsequently, 0.4 mL of 10% (v/v) Na_2_CO_3_ solution was added, and the final volume was adjusted to 4 mL with 0.6 mL of deionized water. After incubation for 1 h at room temperature, the absorbance was measured at 725 nm using a spectrophotometer (UV-1800–240 V, Shimadzu Corporation, Kyoto, Japan). The TPC was calculated using a gallic acid calibration curve and expressed as mg CE g^−1^ dry weight (dw).

#### Determination of total flavonoid content

The total flavonoid content (TPC) was determined following the method of Ghimeray et al. (2014) with minor modifications. Briefly, 0.5 mL of the sample extract was mixed with 0.1 mL of 10% aluminum nitrate and 0.1 mL of 1 M potassium acetate. The mixture was then diluted to a final volume of 4 mL with 3.3 mL of 80% methanol, vortexed, and incubated for 40 minutes. Absorbance was recorded at 415 nm using a spectrophotometer (UV-1800–240 V, Shimadzu Corporation, Kyoto, Japan). The TF content was expressed as milligrams of coumarin equivalents per gram of dry weight (mg CE g^−1^ dry weight, dw).

### Amino acid analysis

Total amino acid content of basil and parsley was determined using the ninhydrin–citrate buffer method (Moore and Stein, 1954). A citrate buffer (pH 5.5) was prepared from citric acid monohydrate and trisodium citrate dihydrate, and a fresh ninhydrin reagent was made by dissolving 2 g of ninhydrin in 100 mL of Dimethyl sulfoxide (DMSO) and diluting to volume with the citrate buffer. For the assay, 1.0 mL of each sample solution was transferred to a clean test tube, followed by the addition of 1.0 mL of ninhydrin reagent. The tubes were mixed thoroughly and heated in a water bath (at 80°C) for 10 minutes to allow color development. After heating, the tubes were cooled to room temperature for 5 minutes, and 2.0 mL of citrate buffer was added to each tube, bringing the total volume to 4.0 mL. The absorbance of the solution was measured at 570 nm against a reagent blank within 30 minutes using a UV-5000 vis-spectrophotometer (UV-1800–240 V, Shimadzu Corporation, Kyoto, Japan). A standard calibration curve was prepared by plotting absorbance against amino acid concentration, and the amino acid content was expressed as mg g^-1^dw.

### Total nitrate content analysis

Nitrate content in the basil and parsley was determined using the salicylic acid method as described by Cataldo et al. (1975) with minor modifications. For the assay, 0.2 mL of the extract was mixed with 0.8 mL of 5% (w/v) salicylic acid prepared in concentrated sulfuric acid (H_2_SO_4_). The mixture was left at room temperature for 20 min to develop color. Subsequently, 19 mL of 2 N sodium hydroxide (NaOH) was carefully added to neutralize the reaction and bring it to completion. After cooling to room temperature, the absorbance was measured at 410 nm using a UV-5000 vis-spectrophotometer (UV-1800–240 V, Shimadzu Corporation, Kyoto, Japan). A standard calibration curve was prepared using potassium nitrate (KNO_3_) solutions ranging from 0 to 100 µgmL^−1^. Nitrate concentration in the samples was calculated from the standard curve and expressed as milligrams of nitrate per gram of dry weight (mg NO_3_^−^ g^−1^ dw).

### Antioxidant analysis

#### DPPH free radical scavenging capacity

The antioxidant capacity of basil and parsley was determined based on the scavenging activity of the stable 2,2-diphenyl-1 picryl hydrazyl (DPPH) free radical according to the methods described by Braca et al., 2003. The DPPH solution was prepared (5.914 mg of DPPH powder dissolved in 100 mL of 100% methanol) to maintain an absorbance range of 1.1–1.3 using a spectrophotometer (UV-1800, 240 V, Shimadzu Corporation, Kyoto, Japan). Briefly, 1 mL of stock solution was added to 3 mL of DPPH solution. The blank sample was prepared by adding 1 mL of methanol instead of the stock extract to 3 mL of DPPH solution. The mixture was shaken vigorously and left to stand at room temperature in the dark for 30 min. The absorbance was measured at 517 nm using a spectrophotometer (UV-1800–240 V, Shimadzu Corporation, Kyoto, Japan). The percentage inhibition activities of the plant samples were calculated against a blank sample using the following equation:


Inhibition(%)=[(bank sample−extract sample)/bank sample] × 100

#### Ferric reducing antioxidant power assay

The reducing power of the basil and parsley was estimated according to the FRAP assay as described by Pulido et al. (2000). In brief, 1 mL of the extracted plant sample was mixed with 1 mL of 0.2 M phosphate buffer to maintain a pH of 6.6. The mixture was then incubated at 50°C for 20 min. After incubation, 1 mL of trichloroacetic acid (TCA) was added to the solution, and the mixture was then centrifuged at 3000 rpm for 10 min. The collected supernatant was diluted with distilled water at a 1:1 ratio. Finally, 0.25 mL of 0.1% ferric chloride was added, and the absorbance was measured at 700 nm by a spectrophotometer (UV-1800–240 V, Shimadzu Corporation, Kyoto, Japan).

#### Chlorophyll and carotenoid determination

For the determination of chlorophyll and carotenoid pigments, the fresh leaves (50 mg) of basil and parsley from each treatment (twelve plants/treatment) were extracted (10 mL of 80% acetone) and placed at room temperature for 30 min. The collected extract was transferred into a tube and centrifuged at 4000 rpm for 10 min. The absorbance was taken at 647, 663, and 470 nm, respectively, using a UV-5000 vis-spectrophotometer (UV-1800–240 V, Shimadzu Corporation, Kyoto, Japan). Chlorophyll *a*, Chlorophyll *b*, and Carotenoid were determined according to the formula proposed by Lichtenthaler (1987) and expressed as mg g^−1^ fresh weight, fw:


$$\text{Chl a}=12.25\;\times \;{\text{A}}_{663}-\;2.79\;\times \;{\text{A}}_{647}$$

$$\text{Chl b}=21.50\;\times \;{\text{A}}_{647}-\;5.10\;\times \;{\text{A}}_{663}$$

$$\text{Car }=\;\left(1000\;\times \;{\text{A}}_{470}-\;1.82\;\times \text{ Chl a}-\;85.02\;\times \text{ Chl b}\right)/198$$

#### Anthocyanin determination

Total anthocyanins of basil and parsley were analyzed according to the modified protocol described by Revilla et al. (1998). Each fresh leaf sample (30 mg) was dissolved in 5 mL of 2% HCl in methanol for 36 h at 4°C, then centrifuged (1446 × g for 15 min), and the supernatant was separated and used as a stock solution for anthocyanins analysis. Following that, 400 mL of the collected supernatant was diluted with 2 mL potassium chloride buffer (0.025 M, pH 1.0) and sodium acetate buffer (0.025 M, pH 1.0). (0.4 M, pH 4.5). After 15 min, each solution was filtered, and absorbance was measured at 515 nm; the maximum absorbance was confirmed in separate scans using a UV-5000 vis-spectrophotometer (UV-1800, 240 V, Shimadzu Corporation, Kyoto, Japan). Total anthocyanins were calculated as the equivalent value of cyaniding-3-glucoside and expressed as mg 100 g^−1^ fw.

### Statistical analysis

Data was analyzed using a one-way ANOVA in Minitab, LLC (version 22.4.0. State College, PA, USA. All data were expressed as mean ± SD. The results obtained under different light treatments were compared using analysis of variance (ANOVA), and mean separations were performed using Fisher’s least significant difference (LSD) test at P ≤ 0.05. Principal component analysis (PCA) was performed using IBM SPSS Statistics software (Version 31.0.1.0; IBM Corp., Armonk, NY, USA) to evaluate species-specific physiological and biochemical responses to different light spectra and irradiance levels. Prior to analysis, all variables were standardized, and principal components were extracted from the eigenvalues to visualize treatment-separation patterns and variable associations.

## Result

### Plant growth characteristics and photochemical activity

Plant morphology and biomass accumulation in basil were significantly affected by FR and UV-A compared with RBW at the same light intensity (**Table 2**). At 25 µmol m^−2^ s^−1^, FR (T2 vs. T1) increased plant height, leaf number, leaf area, fresh weight, and dry weight by approximately 112%, 40%, 194%, 118%, and 329%, respectively. The addition of UV-A, together with FR (T3 vs. T1), further increased leaf area, fresh weight, and dry weight by approximately 656%, 176%, and 471%, respectively.

**Table 2:** Growth characteristics of basil plant grown under different light intensities and spectral compositions.

Treatment	Plant height (cm)	Leaf No.	Leaf area (cm²)	Root length (cm)	Fresh weight (g)	Dry weight (g)
T1	12.5 ± 3 e	10 ± 2 e	3.4 ± 2 f	14.1 ± 3 c	1.7 ± 1 e	0.07 ± 0.1 f
T2	26.5 ± 5 ab	14 ± 4 d	10 ± 3 e	16.7 ± 3 bc	3.7 ± 1 d	0.30 ± 0.1 e
T3	21.5 ± 4 c	12 ± 3 de	25.7 ± 2 a	16.3 ± 3 bc	4.7 ± 1 cd	0.40 ± 0.1 d
T4	11 ± 3 e	11 ± 2 e	11 ± 2 e	20 ± 5 a	1.0 ± 0.4 e	0.05 ± 0.1 f
T5	25 ± 4 b	22 ± 3 b	17 ± 2 c	19 ± 4 ab	4.9 ± 1 d	0.35 ± 0.1 e
T6	25 ± 5 b	25 ± 5 a	26 ± 3 a	18 ± 5 ab	6.8 ± 2 b	0.48 ± 0.3 c
T7	22 ± 3 c	22 ± 2 b	14 ± 5 d	15 ± 4 c	5.7 ± 2 c	0.50 ± 0.2 c
T8	29 ± 4 a	26 ± 2 a	24 ± 3 b	17 ± 5 c	8.0 ± 2 a	0.70 ± 0.3 a
T9	20 ± 4 c	19 ± 3 c	20 ± 2 c	14 ± 3 c	5.8 ± 2 c	0.40 ± 0.2 d
LSD (0.05)	4.23	3.62	4.85	3.41	1.52	0.08

Values are presented as means ± standard deviation (SD). Different letters within each column indicate significant differences among treatments according to Fisher’s least significant difference (LSD) test at P ≤ 0.05.

At 50 µmol m^−2^ s^−1^, FR light (T5 vs. T4) markedly improved shoot growth and biomass accumulation, increasing plant height by 127%, leaf number by 100%, fresh weight by 390%, and dry weight by 600% relative to the RBW. Similarly, the combined FR+UV-A treatment (T6 vs. T4) promoted leaf production, leaf area, and biomass accumulation, resulting in increases of approximately 136%, 136%, 580%, and 860% in leaf number, fresh weight, and dry weight, respectively. Under 100 µmol m^−2^ s^−1^, FR (T8 vs. T7) enhanced plant height, leaf number, leaf area, fresh weight, and dry weight by approximately 32%, 18%, 71%, 40%, and 40%, respectively. In contrast, the addition of UV-A together with FR (T9 vs. T7) reduced plant height and biomass accumulation compared with FR alone.

Plant growth and biomass accumulation in parsley responded differently to FR and UV-A depending on light intensity (**Table 3**). At 25 µmol m^−2^ s^−1^, FR (T2 vs. T1) increased plant height and leaf area by approximately 15% and 33%, respectively, but reduced fresh and dry weight by about 20% and 33%. The addition of UV-A together with FR (T3 vs. T1) markedly reduced plant height, leaf area, root length, fresh weight, and dry weight compared with the RBW.

**Table 3:** Growth characteristics of the parsley plant grown under different light intensities and spectral compositions.

Treatment	Plant height (cm)	Leaf no.	Leaf area (cm²)	Root length (cm)	Fresh weight (g)	Dry weight (g)
T1	13.0 ± 3 bc	4 ± 0 a	9.0 ± 3 c	18 ± 4 bc	1.5 ± 0.3 b	0.15 ± 0.1c
T2	15.0 ± 4 a	4 ± 0 a	12 ± 3 b	17 ± 4 bc	1.2 ± 0.2 c	0.10 ± 0.2d
T3	5.5 ± 2 e	4 ± 0 a	4 ± 2 e	14 ± 5 c	1.0 ± 0.2 d	0.12 ± 0.1d
T4	12.5 ± 3 c	4 ± 0 a	13.3 ± 3 b	21 ± 6 a	1.2 ± 0.1 c	0.16 ± 0.1c
T5	14.3 ± 3 b	4 ± 0 a	26.0 ± 0 a	19 ± 5 ab	1.8 ± 0.3 a	0.21 ± 0.1 b
T6	7.5 ± 2 d	4 ± 0 a	7.8 ± 2 d	20 ± 4 ab	0.15 ± 0.1 e	0.75 ± 0.2 a
T7	14.3 ± 3 b	4.5 ± 1 a	10 ± 4 c	16.5 ± 3 c	1.6 ± 0.1 b	0.21 ± 0.2b
T8	11.3 ± 2 c	5.0 ± 0 a	9.7 ± 2 c	17.6 ± 4 bc	1.9 ± 0.2 a	0.22 ± 0.1b
T9	6.3 ± 2 e	5.0 ± 0 a	8.7 ± 2 cd	20 ± 6 ab	1.9 ± 0.3 a	0.20 ± 0.1b
LSD (0.05)	2.54	NS	3.76	3.85	0.19	0.03

Values are presented as means ± standard deviation (SD). Different letters within each column indicate significant differences among treatments according to Fisher’s least significant difference (LSD) test at P ≤ 0.05.

At 50 µmol m^−2^ s^−1^, FR (T5 vs. T4) substantially enhanced leaf area, fresh weight, and dry weight by approximately 95%, 50%, and 31%, respectively, compared with the RBW. In contrast, the combined FR+UV-A(T6 vs. T4) reduced plant height, leaf area, and fresh weight, although dry weight increased considerably. Root length remained relatively stable among T4, T5, and T6. Under 100 µmol m^−2^ s^−1^, FR(T8 vs. T7) slightly increased fresh and dry biomass, while plant height decreased relative to the RBW. Similarly, the combined FR+UV-A (T9 vs. T7) maintained relatively high fresh and dry weight but substantially reduced plant height.

Total phenolic content (TPC) and total flavonoid content (TFC) were significantly influenced by FR and UV-A in both basil and parsley compared with RBW at the same light intensity (**Table 4**). In basil, the addition of FR at 25 µmol m^−2^ s^−1^ (T2 vs. T1) increased TPC and TFC by approximately 55% and 35%, respectively, while the addition of FR+UV-A (T3 vs. T1) increased TPC by 29% and TFC by 12%. At 50 µmol m^−2^ s^−1^, FR (T5 vs. T4) enhanced TPC by 20% and markedly increased TFC by 98%, whereas FR+UV-A (T6 vs. T4) reduced TPC by 10% and TFC by 9% compared with FR alone. Under 100 µmol m^−2^ s^−1^, FR addition (T8 vs. T7) increased TPC and TFC by 32% and 28%, respectively, while FR+UV-A (T9 vs. T7) increased TPC by 17% but reduced TFC by 34%.

**Table 4:** Total phenolic and total flavonoid content of basil and parsley grown under different light intensities and spectral compositions.

Plant species	Treatment	TPC (mgg^-1^ dw)	TFC (mgg^-1^ dw)
Basil	T1	12.5 ± 0.5d	3.4 ± 0.4d
	T2	19.4 ± 0.9a	4.6 ± 0.5c
	T3	16.1 ± 0.7b	3.8 ± 0.4d
	T4	16.5 ± 0.8b	4.5 ± 1.0c
	T5	19.8 ± 0.6a	8.9 ± 0.9a
	T6	14.8 ± 0.6c	4.1 ± 0.8c
	T7	13.8 ± 0.7c	6.5 ± 1.1b
	T8	18.2 ± 0.8a	8.3 ± 1.2a
	T9	16.2 ± 1.0b	4.3 ± 1.5c
	LSD <0.05	1.42	1.96
Parsley	T1	11.3 ± 0.5f	12.8 ± 0.8c
	T2	11.2 ± 0.4f	10.5 ± 0.7c
	T3	13.5 ± 0.7e	19.0 ± 1.3a
	T4	12.0 ± 0.6ef	13.2 ± 0.9c
	T5	14.0 ± 0.8de	16.0 ± 1.0b
	T6	14.0 ± 0.7de	18.3 ± 1.2a
	T7	16.9 ± 0.9c	12.8 ± 0.8c
	T8	18.7 ± 1.0b	18.4 ± 1.1a
	T9	22.0 ± 1.3a	18.9 ± 1.4a
	LSD <0.05	1.58	2.04

Values are presented as means ± standard deviation (SD). Different letters within each column indicate significant differences among treatments according to Fisher’s least significant difference (LSD) test at P ≤ 0.05.

TPC, Total phenolic content; TFC, Total flavonoid content.

In parsley, FR at 25 µmol m^−2^ s^−1^ (T2 vs. T1) had minimal effect on TPC and reduced TFC by approximately 18%, whereas FR+UV-A (T3 vs. T1) increased TPC and TFC by about 19% and 48%, respectively. At 50 µmol m^−2^ s^−1^, FR (T5 vs. T4) increased TPC by 17% and TFC by 21%, while FR+UV-A (T6 vs. T4) increased TPC by 17% and TFC by 39%. Under 100 µmol m^−2^ s^−1^, FR addition (T8 vs. T7) enhanced TPC and TFC by approximately 11% and 44%, respectively, while FR+UV-A (T9 vs. T7) further increased TPC by 30% and TFC by 48%.

Photosynthetic pigment content was significantly influenced by FR and UV-A in both basil and parsley compared with RBW (**Table 5**). In basil, FR at 25 µmol m^−2^ s^−1^ (T2 vs. T1) increased Chl *a*, Chl *b*, and carotenoids by approximately 10%, 12%, and 13%, respectively, whereas the addition of UV-A (T3 vs. T1) reduced these pigments by 24%, 22%, and 23%. At 50 µmol m^−2^ s^−1^, FR (T5 vs. T4) decreased Chl *a*, Chl *b*, and carotenoids by about 40%, 43%, and 20%, respectively, while FR+UV-A (T6 vs. T4) also reduced pigment accumulation compared with the RBW. In contrast, under 100 µmol m^−2^ s^−1^, FR (T8 vs. T7) markedly enhanced Chl *a*, Chl *b*, and carotenoids by approximately 72%, 67%, and 66%, respectively. Similarly, FR+UV-A (T9 vs. T7) increased Chl *a*, Chl *b*, and carotenoids by about 55%, 50%, and 52%, respectively.

**Table 5:** Chlorophyll and carotenoid content of basil and parsley grown under different light intensities and spectral compositions.

Plant species	Treatment	Chl *a* (mg g^-1^ fw)	Chl *b* (mg g^-1^ fw)	Carotenoid (mg g^-1^ fw)
Basil	T1	459 ± 48 c	213 ± 60 d	102 ± 21 c
	T2	503 ± 71 bc	239 ± 42 c	115 ± 34 bc
	T3	349 ± 57 d	167 ± 47 e	79 ± 26 d
	T4	620 ± 127 ab	306 ± 94 b	121 ± 36 bc
	T5	372 ± 55 d	175 ± 64 e	97 ± 31 c
	T6	429 ± 74 c	191 ± 65 de	87 ± 24 d
	T7	397 ± 65 d	214 ± 71 d	101 ± 32 c
	T8	681 ± 124 a	357 ± 112 a	168 ± 56 a
	T9	615 ± 133 ab	321 ± 88 ab	154 ± 23 ab
	LSD (0.05)	153.98	127.94	56.59
Parsley	T1	296 ± 58 bc	106 ± 33 c	33 ± 12 e
	T2	332 ± 66 ab	135 ± 28 bc	77 ± 16 ab
	T3	265 ± 52 c	116 ± 45 c	49 ± 16 cd
	T4	318 ± 76 ab	168 ± 40 ab	45 ± 14 d
	T5	291 ± 84 bc	137 ± 46 bc	78 ± 17 ab
	T6	94 ± 26 e	12 ± 5 e	76 ± 11 ab
	T7	283 ± 77 bc	127 ± 42 bc	44 ± 16 d
	T8	240 ± 47 c	113 ± 23 c	83 ± 21 a
	T9	363 ± 89 a	187 ± 76 a	63 ± 20 bc
	LSD (0.05)	114.35	71.67	27.77

Values are presented as means ± standard deviation (SD). Different letters within each column indicate significant differences among treatments according to Fisher’s least significant difference (LSD) test at P ≤ 0.05.

In parsley, pigment responses to FR and UV-A varied depending on light intensity and pigment type. At 25 µmol m^−2^ s^−1^, FR (T2 vs. T1) moderately enhanced Chl *a*, Chl *b*, while carotenoid content increased substantially. The addition of UV-A (T3 vs. T1) slightly decreased chlorophyll accumulation but maintained higher carotenoid levels than the RBW. Under 50 µmol m^−2^ s^−1^, FR (T5 vs. T4) maintained chlorophyll content near the control level while markedly increasing carotenoids. However, combining FR with UV-A (T6 vs. T4) caused a sharp decline in chlorophyll pigments, particularly Chl *b*, despite relatively stable carotenoid accumulation. At 100 µmol m^−2^ s^−1^, FR (T8 vs. T7) primarily enhanced carotenoid content, whereas the combined FR+UV-A treatment (T9 vs. T7) promoted both Chl *a*, Chl *b* accumulation.

The interaction of FR and UV-A significantly altered chlorophyll fluorescence and stomatal conductance (g_s_)in both basil and parsley, although the response patterns differed between species (**Table 6**). In basil, the strongest photochemical performance was observed under FR at 25 µmol m^−2^ s^−1^), (T2), where *F*_v_/*F*_m_ and PI increased markedly compared with the RBW (T1). At 50 µmol m^−2^ s^−1^, both FR (T5) and FR+UV-A (T6) maintained relatively high g_s_, with the highest g_s_ recorded under T6, although *F*_v_/*F*_m_ indices declined compared with the RBW. Under 100 µmol m^−2^ s^−1^, the RBW with no FR or UV-A (T7) maintained comparatively higher PI values, while the addition of FR (T8) or FR+UV-A (T9) reduced *F*_v_/*F*_m_.

**Table 6:** Maximum quantum yield and stomatal conductance of basil and parsley grown under different light intensities and spectral compositions.

Plant species	Treatment	*F*_v_/*F*_m_	PI	g_s_
Basil	T1	0.73 ± 0.2ab	0.98 ± 0.2c	222 ± 69c
	T2	0.81 ± 0.2a	3.00 ± 0.8a	134 ± 24d
	T3	0.60 ± 0.2c	0.55 ± 0.2d	183 ± 38c
	T4	0.69 ± 0.2b	0.78 ± 0.2cd	298 ± 76ab
	T5	0.68 ± 0.2b	0.41 ± 0.1e	288 ± 64ab
	T6	0.65 ± 0.2bc	0.71 ± 0.1cd	327 ± 77a
	T7	0.79 ± 0.2a	1.80 ± 0.4b	212 ± 32c
	T8	0.69 ± 0.2b	0.60 ± 0.1d	141 ± 34 d
	T9	0.63 ± 0.1c	0.37 ± 0.1e	192 ± 33 c
	**LSD (0.05)**	**0.10**	**0.52**	**71.5**
Parsley	T1	0.78 ± 0.02 a	1.16 ± 0.4 b	89 ± 11 d
	T2	0.68 ± 0.02 bc	0.33 ± 0.01 de	92 ± 22 d
	T3	0.77 ± 0.02 ab	1.64 ± 0.1 a	65 ± 17 e
	T4	0.68 ± 0.02 bc	1.68 ± 0.3 a	94 ± 13 cd
	T5	0.77 ± 0.02 ab	0.71 ± 0.02 c	143 ± 26 b
	T6	0.75 ± 0.02 ab	1.33 ± 0.4 b	118 ± 17 bc
	T7	0.69 ± 0.03 bc	0.45 ± 0.02 d	259 ± 76 a
	T8	0.69 ± 0.03 bc	0.70 ± 0.02 c	143 ± 46 b
	T9	0.65 ± 0.02 c	0.17 ± 0.01 e	162 ± 37 b
	**LSD (0.05)**	**0.09**	**0.36**	**39.8**

Values are presented as means ± standard deviation (SD). Different letters within each column indicate significant differences among treatments according to Fisher’s least significant difference (LSD) test at P ≤ 0.05.

Fv/Fm, Maximum quantum yield; PI, Performance index; gs, Stomatal conductance; LSD, Least significant difference.

Parsley exhibited a contrasting response pattern. The highest *F*_v_/*F*_m_ was recorded under the RBW (T1), while PI reached maximum values under T3 and T4. FR supplementation at 50 and 100 µmol m^−2^ s^−1^ substantially enhanced stomatal conductance, particularly under T7, which showed nearly a threefold increase in g_s_ compared with T1.

**Figure 1:**
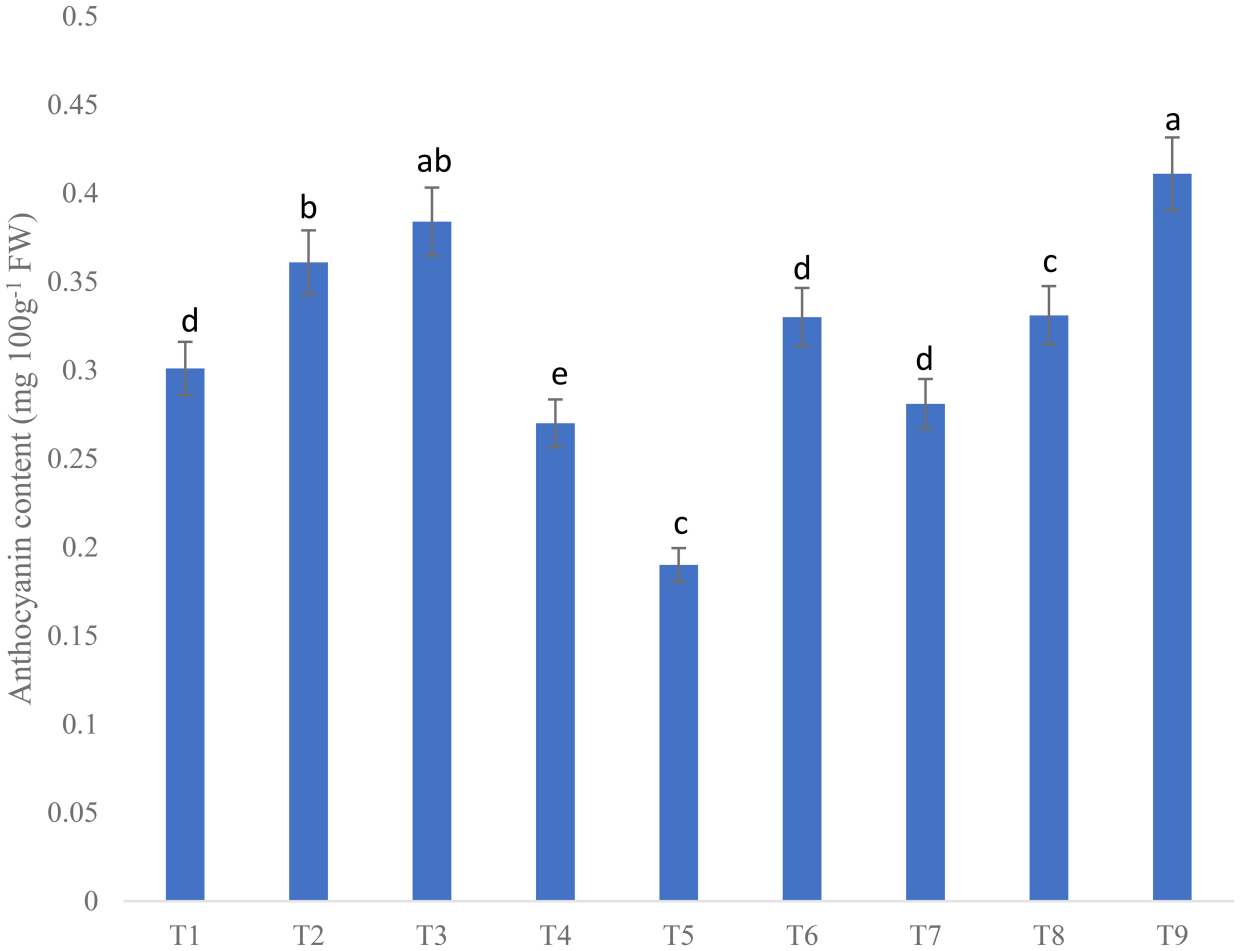
Effects of supplemental FR and UVA light on anthocyanin content of basil. Bars represent means ± SD. Different letters within each bar indicate significant differences among treatments according to Fisher’s least significant difference (LSD) test at P ≤ 0.05.

Anthocyanin content in basil was significantly influenced by the treatments (**Figure 1**). Compared with the RBW at the same light intensity, FR increased anthocyanin accumulation under 25 and 100 µmol m^−2^ s^−1^. At 25 µmol m^−2^ s^−1^, FR (T2 vs. T1) increased anthocyanin content by approximately 20%, while the combined FR+UV-A (T3 vs. T1) further enhanced anthocyanin accumulation by about 27%. In contrast, under 50 µmol m^−2^ s^−1^, FR alone (T5 vs. T4) reduced anthocyanin content by approximately 30%, whereas the addition of UV-A (T6 vs. T4) restored and increased anthocyanin accumulation by nearly 22% compared with the control. Under 100 µmol m^−2^ s^−1^, FR (T8 vs. T7) increased anthocyanin content by approximately 18%, while the combined FR+UV-A (T9 vs. T7) produced the highest anthocyanin accumulation, showing nearly 46% higher values than the RBW.

**Figure 2:**
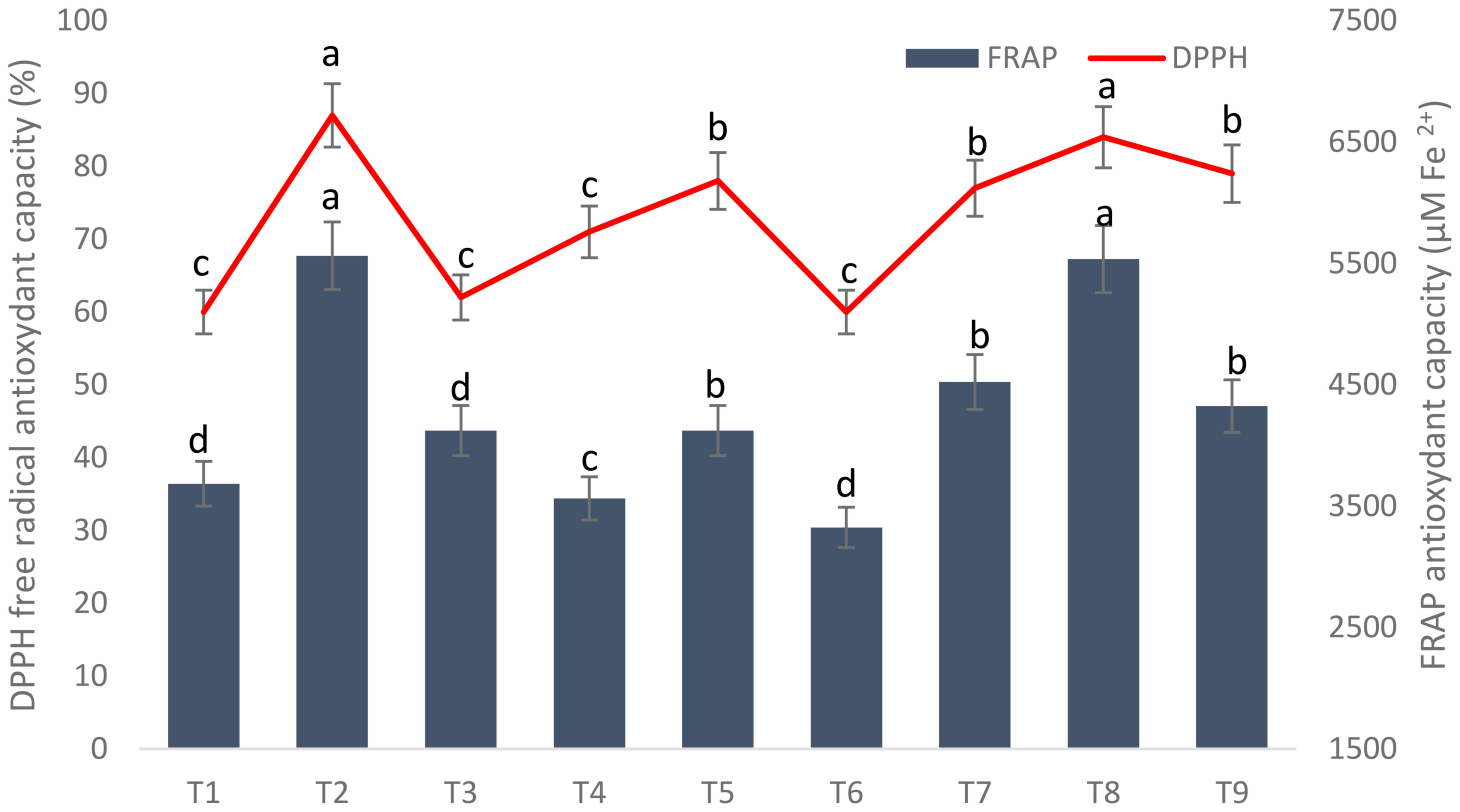
Effects of FR and UVA on antioxidant capacity of basil. DPPH antioxidant capacity (%) (Bar graph) and FRAP (line graph). Bars represent SD. Different letters within each bar indicate significant differences among treatments according to Fisher’s least significant difference (LSD) test at P ≤ 0.05.

The antioxidant capacity of basil varied considerably depending on light intensity and spectral composition (**Figure 2**). Both DPPH radical scavenging activity and FRAP values generally increased following FR compared with the corresponding RBW. At 25 µmol m^−2^ s^−1^, the inclusion of FR (T2) produced a pronounced enhancement in antioxidant activity relative to T1. Under 50 µmol m^−2^ s^−1^, antioxidant capacity remained relatively higher under FR (T5), while the addition of UV-A (T6) resulted in lower DPPH and FRAP responses. A different pattern was observed at 100 µmol m^−2^ s^−1^, where FR (T8) exhibited the strongest antioxidant potential among all treatments, as reflected by the highest FRAP activity and elevated DPPH scavenging capacity. The combined FR+UV-A (T9) also maintained comparatively high antioxidant activity, although it was slightly lower than that of FR alone.

**Figure 3:**
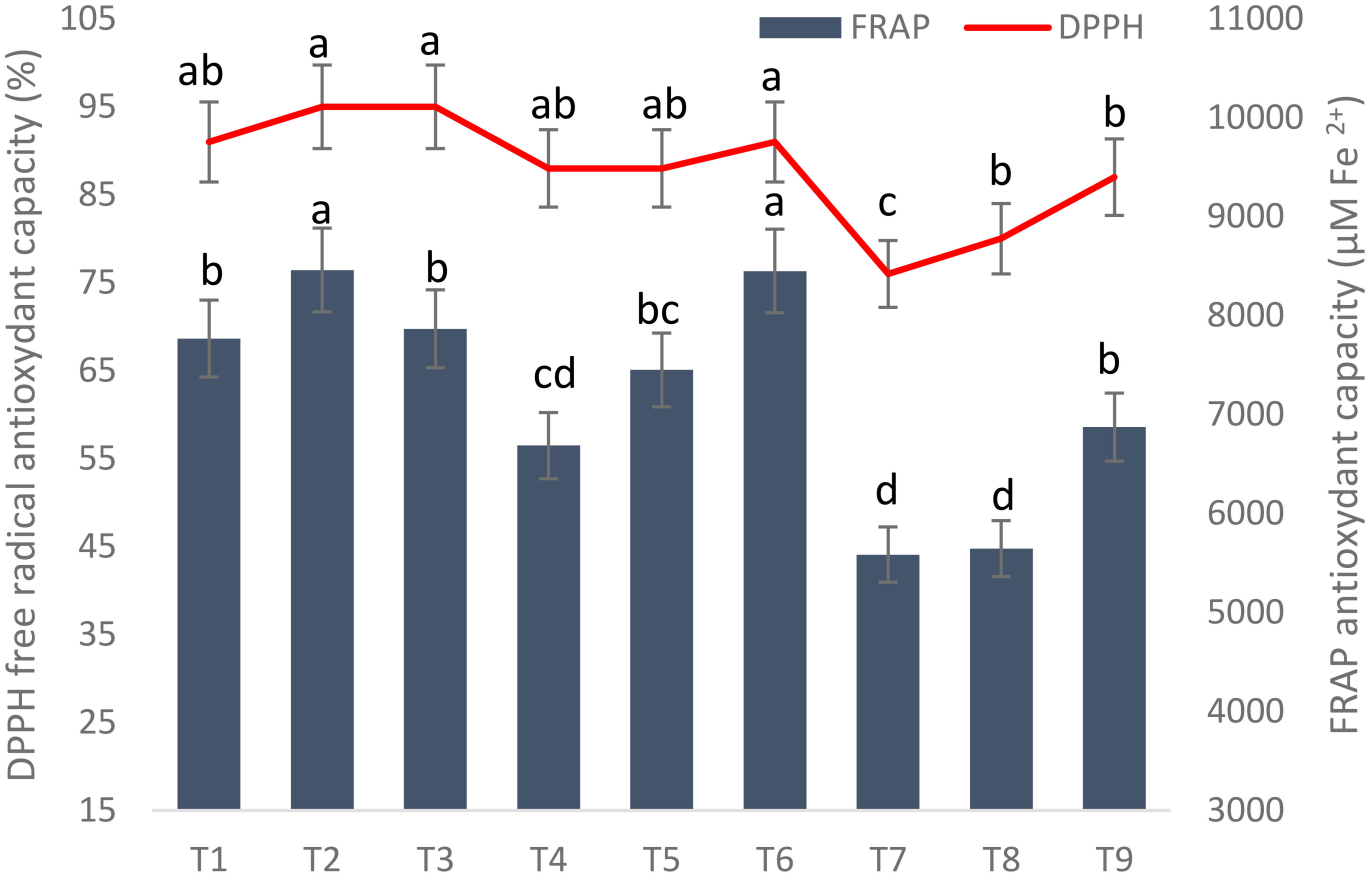
Effects of FR and UVAon antioxidant capacity of parsley. DPPH antioxidant capacity (%) (Bar graph) and FRAP (line graph). Bars represent ± SD. Different letters within each bar indicate significant differences among treatments according to Fisher’s least significant difference (LSD) test at P ≤ 0.05.

In parsley, antioxidant activity was also strongly influenced by interactions of FR, and UV-A (**Figure 3**). Under 25 µmol m^−2^ s^−1^, both FR (T2) and FR+UV-A (T3) enhanced antioxidant potential compared with the RBW (T1), with T2 showing the highest FRAP activity while maintaining elevated DPPH scavenging capacity. At 50 µmol m^−2^ s^−1^, the combined FR+UV-A (T6) produced the strongest antioxidant response, exhibiting the highest FRAP value together with consistently high DPPH activity. In contrast, the RBW at the same intensity (T4) showed comparatively lower antioxidant capacity. At 100 µmol m^−2^ s^−1^, antioxidant activity declined substantially under FR alone (T8) and the RBW (T7), particularly for FRAP activity. However, the addition of UV-A together with FR (T9) partially restored antioxidant capacity, resulting in higher DPPH and FRAP values than T7 and T8.

**Figure 4:**
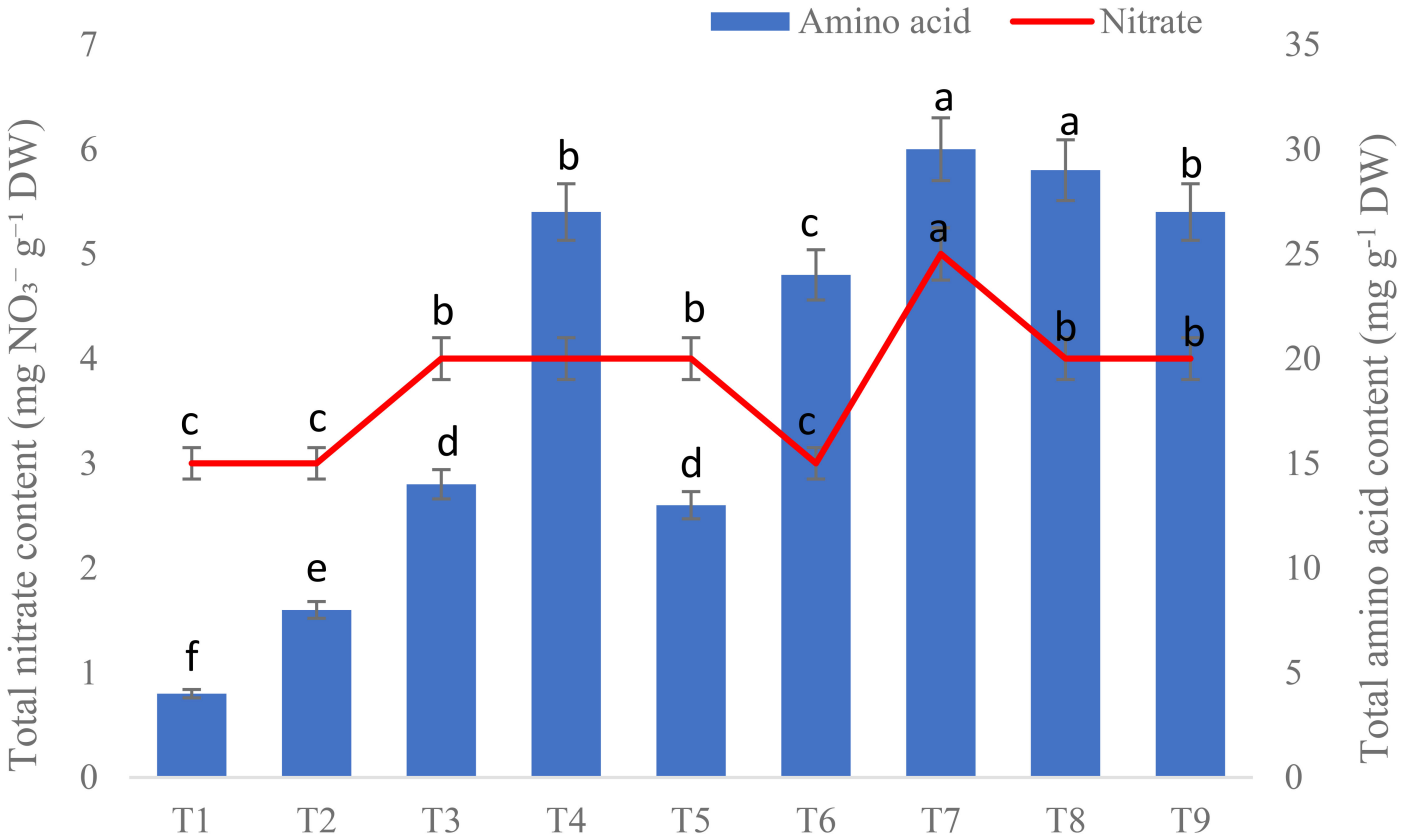
Effects of supplemental FR and UVA light on total nitrate (line graph) and total amino acid (bar graph) content in basil. Bars represent ± SD. Different letters within each bar indicate significant differences among treatments according to Fisher’s least significant difference (LSD) test at P ≤ 0.05.

The accumulation of nitrate and amino acids in basil was markedly affected by spectral composition and light intensity (**Figure 4**). Under 25 µmol m^−2^ s^−1^, FR (T2) slightly increased amino acid concentration relative to the RBW (T1), whereas the combined FR+UV-A (T3) produced a stronger stimulatory effect on both nitrate and amino acid accumulation. At 50 µmol m^−2^ s^−1^, FR (T5) maintained nitrate content like T3 but reduced amino acid accumulation compared with the RBW (T4). In contrast, the addition of UV-A together with FR (T6) substantially enhanced amino acid concentration while lowering nitrate content relative to T4 and T5. The RBW (T7) exhibited the highest nitrate concentration and among the highest amino acid levels. Although FR alone (T8) slightly reduced nitrate accumulation compared with T7, amino acid concentration remained elevated. Similarly, the combined FR+UV-A (T9) maintained high amino acid content while nitrate levels remained comparatively stable.

**Figure 5:**
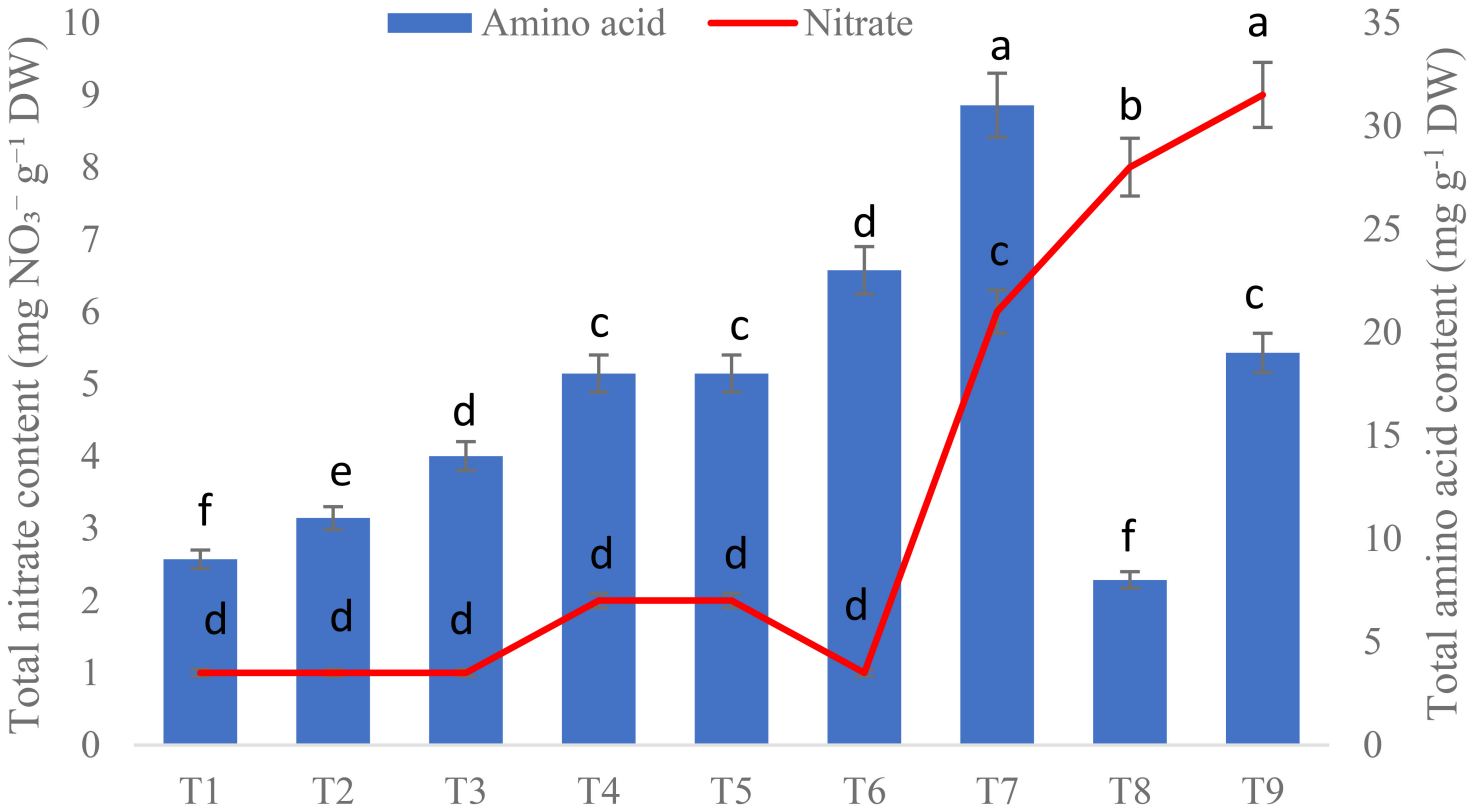
Effects of supplemental FR and UVA light on total nitrate (line graph) and total amino acid (bar graph) content in parsley. Bars represent ± SD. Different letters within each bar indicate significant differences among treatments according to Fisher’s least significant difference (LSD) test at P ≤ 0.05.

In parsley, nitrate and amino acid accumulation showed distinct responses to FR and UV-A across different light intensities (**Figure 5**). At 25 µmol m^−2^ s^−1^, nitrate content remained relatively unchanged among T1, T2, and T3, whereas amino acid concentration gradually increased following the addition of FR and FR+UV-A. At 50 µmol m^−2^ s^−1^, both FR (T5) and FR+UV-A (T6) treatments enhanced amino acid accumulation compared with the RBW (T4), with T6 showing the strongest response. A contrasting trend was observed under 100 µmol m^−2^ s^−1^. The RBW (T7) exhibited the highest amino acid concentration, along with a sharp increase in nitrate accumulation. FR alone (T8) markedly reduced amino acid content compared with T7, despite maintaining relatively high nitrate levels. In contrast, the addition of UV-A together with FR (T9) partially restored amino acid accumulation while further increasing nitrate concentration to the highest level among all treatments.

**Figure 6:**
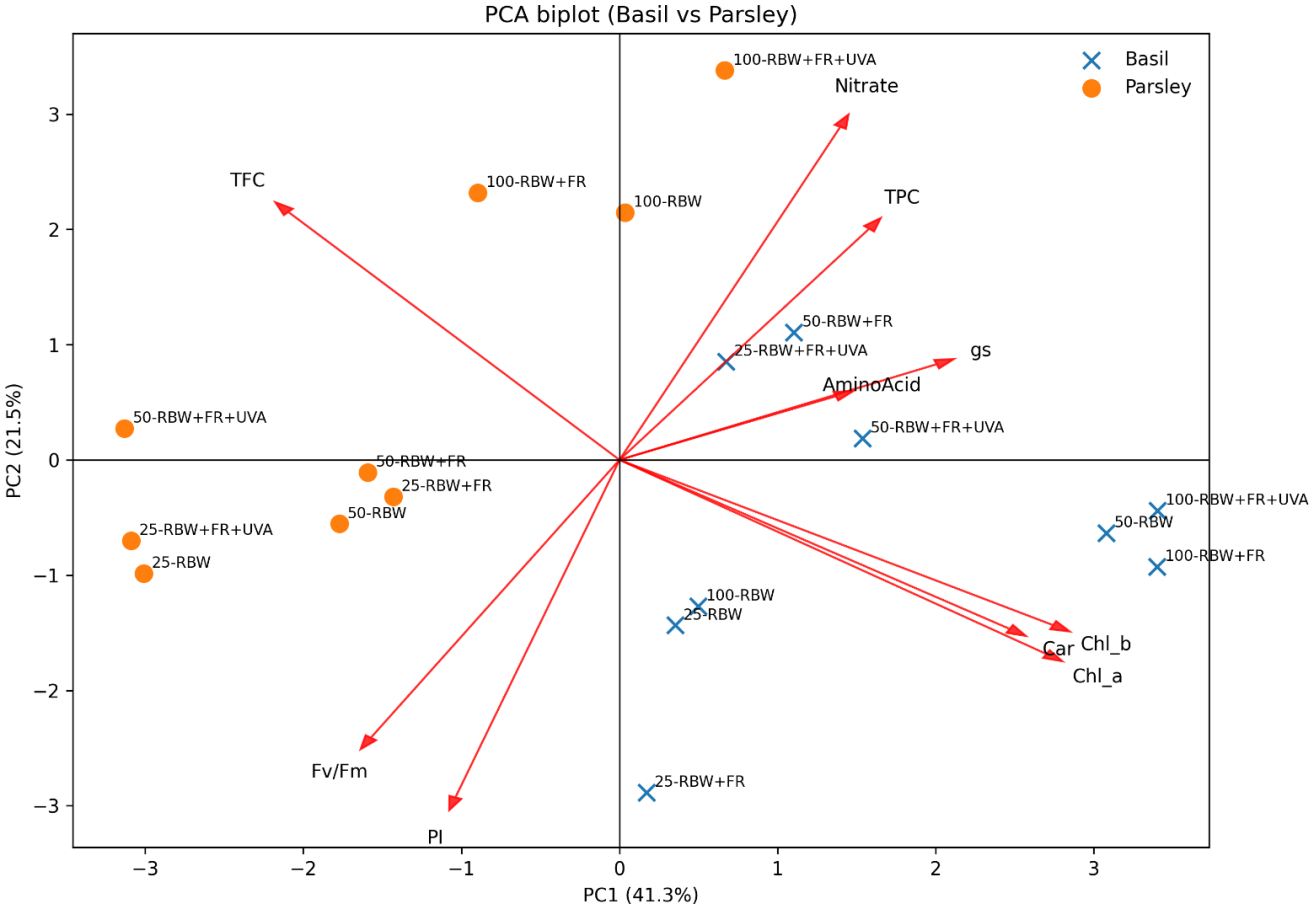
Principal component analysis (PCA) biplot illustrating the multivariate relationships among photosynthetic traits, pigment composition, nitrogen metabolites, and antioxidant properties in basil and parsley grown under different light spectra and PPFD levels. The PCA biplot illustrates the distribution of basil (×) and parsley (●) grown under RBW, RBW+FR, and RBW+FR+UVA at 100, 50, and 25 µmol m^−2^ s^−1^. PC1 reflects nitrogen metabolism and secondary metabolites (amino acids, nitrate, TPC, TFC), while PC2 represents photosynthetic performance and pigment traits (chlorophylls, carotenoids, *F*_v_/*F*_m_, PI, g_s_). Red vectors indicate variable loadings, and sample proximity to vectors denotes trait associations. Species separation indicates distinct acclimation strategies to light spectrum and intensity.

The PCA biplot reveals clear species-specific acclimation strategies to light spectrum and intensity (**Figure 6**). Separation along PC1 indicates a dominant nitrogen–secondary metabolism axis, with positive loadings from amino acids, nitrate, TPC, and TFC. Parsley samples cluster toward positive PC1 values, demonstrating a greater propensity to allocate resources toward nitrogen assimilation and secondary metabolite synthesis, particularly under RBW+FR+UVA, consistent with UVA-driven elicitation of phenolic and flavonoid pathways.

In contrast, PC2 represents a photosynthetic pigment axis, driven by chlorophyll a, chlorophyll b, carotenoids, *F*_v_/*F*_m_, PI, and g_s_. Basil samples predominantly align along PC2, reflecting a strategy centered on optimizing photosynthetic efficiency and pigment accumulation, especially under RBW+FR, which enhanced PI and chlorophyll content. This suggests that FR promotes efficient light harvesting and photochemical performance in basil. The opposing orientation of nitrogen-related variables relative to pigment and photosynthetic traits highlights a carbon–nitrogen trade-off between the two species. While parsley prioritizes nitrogen storage and antioxidant defenses, basil preferentially channels assimilated carbon into photosynthetic machinery. Notably, RBW+FR+UVA shifts parsley toward higher PC1 and PC2 values (enhanced antioxidants with maintained photosynthetic function), whereas the same treatment drives basil downward along PC2 at lower PPFD, indicating photosynthetic stress under UVA when light intensity is limiting.

## Discussion

### Plant growth characteristics

The results of this study indicate that basil and parsley responded differently to light intensity and spectral composition, demonstrating species-dependent acclimation to FR and UV-A (**Tables 2**, **3**). In basil, vegetative growth and biomass accumulation were strongly stimulated by FR, particularly under 50 and 100 µmol m^−2^ s^−1^. The greatest enhancement in plant height, fresh weight, and dry weight under RBW+FR at 100 µmol m^−2^ s^−1^ suggests that FR promoted more efficient canopy development and biomass production when adequate photosynthetic photon flux was available. Similar responses have been reported in leafy vegetables, where FR supplementation increased leaf expansion, shoot elongation, and radiation capture efficiency through phytochrome-mediated regulation of shade-acclimation processes (Fraser et al., 2021; Meng et al., 2024; Zhen and Bugbee, 2020). Increased leaf expansion under FR may enhance whole-canopy light interception, thereby improving carbon assimilation and biomass accumulation in controlled environments.

The addition of UV-A together with FR produced contrasting responses depending on light intensity (He et al., 2021). Under moderate light intensity, the combined FR+UV-A promoted leaf area expansion and biomass accumulation in basil, whereas under 100 µmol m^−2^ s^−1^, UV-A appeared to suppress some of the FR-induced growth responses. UV-A radiation is known to regulate plant morphology through cryptochrome-mediated pathways that influence epidermal cell expansion and photomorphogenic acclimation (Hernández and Kubota, 2020; Rai et al., 2021). However, UV-A under higher irradiance may increase metabolic costs associated with photoprotection and antioxidant defense, thereby limiting further vegetative growth (Pathak et al., 2019). This may explain why biomass accumulation under T9 remained lower than under FR alone (T8).

Root growth patterns were less responsive to spectral quality than shoot-related traits. In both basil and parsley, longer roots were generally observed at 25-50 µmol m^−2^ s^−1^, indicating that light quantity, rather than spectral composition. Carotti et al. (2024), who suggested that plants grown under non-saturating irradiance allocate a proportionally greater share of assimilates to root development to improve resource acquisition efficiency. The limited effect of FR on root elongation further supports the concept that FR primarily enhances shoot competitiveness and canopy expansion rather than root biomass allocation (Wang J. L. et al., 2025).

Compared with basil, parsley exhibited weaker shoot elongation responses to FR, indicating lower sensitivity to phytochrome-regulated shade-avoidance signaling. Nevertheless, parsley responded positively to FR at moderate and high light intensities through increased leaf area and biomass accumulation (De Clercq and Van Labeke, 2021). Interestingly, leaf number remained relatively stable across all treatments, suggesting that leaf initiation in parsley is more developmentally regulated and less influenced by spectral manipulation (Knight, 2025). Similar responses have been reported in Apiaceae species, where environmental factors alter leaf expansion more readily than leaf initiation rate (Litvin et al., 2022). In addition, the reduction in plant height observed under combined FR+UV-A suggests that UV-A partially counteracted FR-induced elongation responses in parsley. Such antagonistic interactions between FR and UV-A have been associated with UV-mediated suppression of excessive stem elongation and enhanced photomorphogenic control (Rai et al., 2021).

Under 25 µmol m^−2^ s^−1^, both species exhibited reduced biomass accumulation, particularly under RBW alone, indicating that the available photon flux was insufficient to sustain optimal photochemical activity and growth. Although FR and UV-A partially improved growth under relatively low irradiance. Previous studies have shown that, while FR can improve light-use efficiency and canopy photon capture, total light intensity remains the dominant factor controlling biomass production in controlled-environment agriculture (Ali et al., 2024; Meng et al., 2024).

### Total phenolics and flavonoids content

The accumulation of phenolic and flavonoid compounds was strongly regulated by light intensity and spectral composition in both basil and parsley. In basil, FR generally enhanced TPC and TFC accumulation, particularly under 25 µmol m^−2^ s^−1^ and 100 µmol m^−2^ s^−1^ light intensities. The increase in TPC under RBW+FR treatments suggests that FR-induced phytochrome signaling stimulated the phenylpropanoid pathway and promoted antioxidant-related secondary metabolism. Previous studies have shown that reduced red:far-red ratios can increase PAL activity and enhance carbon allocation toward phenolic biosynthesis under FR-enriched environments (Fraser et al., 2021; Park and Runkle, 2018). The marked increase in flavonoid concentration under T5 and T8 further indicates that FR supplementation promoted photoprotective metabolic acclimation (Kim and Eom, 2025).

The UV-A produced intensity-dependent responses in basil. Under 25 µmol m^−2^ s^−1^ light, FR+UV-A moderately enhanced TPC relative to the RBW, whereas under 100 µmol m^−2^ s^−1^, UV-A reduced flavonoid accumulation compared with FR alone. Such responses may reflect increased metabolic demand associated with UV-induced stress acclimation or altered carbon partitioning between growth and defense-related pathways. Similar findings were reported by Morales et al. (2010), who demonstrated that UV-A can differentially regulate flavonoid biosynthesis depending on irradiance level and environmental conditions.

In parsley, phenolic and flavonoid accumulation was also significantly influenced by spectral enrichment. The combined FR+UV-A, particularly under 25 µmol m^−2^ s^−1^ and 100 µmol m^−2^ s^−1^, promoted greater accumulation of antioxidant-related compounds. UV-A activates UVR8- and cryptochrome-mediated signaling pathways, thereby inducing transcription factors such as HY5 and stimulating flavonoid biosynthetic genes (Morales et al., 2010; Rai et al., 2021). The elevated TFC observed under T3, T6, T8, and T9 indicates that FR and UV-A together enhanced secondary metabolic activity associated with antioxidant defense.

### Photosynthetic pigment and antioxidant capacity

The responses of photosynthetic pigments to FR and UV-A were strongly dependent on light intensity and spectral interaction (Samuoliene et al., 2020). In basil, the substantial increase in chl *a*, chl *b*, and carotenoid content under FR-enriched spectra at 100 µmol m^−2^ s^−1^ indicates improved light-harvesting capacity and enhanced development of the photosynthetic apparatus. Far-red light has previously been reported to promote chlorophyll accumulation by stimulating leaf expansion and increasing nitrogen allocation toward photosynthetic pigments and antenna complexes (Carotti et al., 2024; Zhen and Bugbee, 2020). The strong enhancement in carotenoid accumulation under relatively high-intensity FR treatments further suggests improved photoprotection and energy dissipation capacity. In contrast, reductions in pigment content observed under FR+UV-A treatments at 25 µmol m^−2^ s^−1^ may indicate that UV-A induced mild oxidative or photoprotective stress, resulting in downregulation of chlorophyll accumulation as part of an acclimation response. Similar UV-mediated pigment adjustments have been reported in leafy crops exposed toUV-A radiation (Morales et al., 2010).

In parsley, pigment accumulation also responded markedly to spectral manipulation, although the response pattern varied with light intensity (Semenova et al., 2023). Chlorophyll accumulation was greatest under combined FR+UV-A treatments at 100 µmol m^−2^ s^−1^, while carotenoid concentration increased strongly under FR-enriched spectra across multiple light levels. Carotenoids function as essential photoprotective compounds involved in non-photochemical quenching and reactive oxygen species (ROS) scavenging(Liu et al., 2023). The enhancement of carotenoid accumulation under FR treatments, therefore, suggests increased activation of photoprotective mechanisms under spectral enrichment.

Anthocyanin accumulation was also highly responsive to interactions among FR, UV-A, and light intensity (Wang F. et al., 2025). The progressive increase in anthocyanin content under FR+UV-A treatments at 100 µmol m^−2^ s^−1^ suggests a synergistic interaction between these wavelengths when sufficient photosynthetic energy was available. Far-red light alters phytochrome photoequilibrium and can promote assimilate allocation toward secondary metabolism, whereas UV-A activates UVR8–HY5 signaling pathways that regulate key anthocyanin biosynthetic genes such as chalcone synthase (CHS) and dihydroflavonol reductase (DFR) (Fraser et al., 2021; Rai et al., 2021). Under 50 µmol m^−2^ s^−1^ light intensity, FR alone reduced anthocyanin accumulation relative to FR+UV-A treatments, suggesting that FR primarily promoted growth-related carbon utilization, while UV-A redirected metabolism toward protective pigment synthesis. At 25 µmol m^−2^ s^−1^ intensity, elevated anthocyanin accumulation under FR- and UV-A-enriched spectra likely reflected photoprotective acclimation in response to reduced photosynthetic capacity and increased excitation pressure (Morales et al., 2010).

The antioxidant responses measured by DPPH and FRAP generally mirrored trends in anthocyanin and phenolic accumulation, indicating that changes in antioxidant capacity were closely associated with secondary metabolite biosynthesis. In basil, FR, particularly at 100 µmol m^−2^ s^−1^, markedly enhanced antioxidant activity, suggesting increased accumulation of antioxidant-related metabolites under FR-enriched conditions. In parsley, the strongest antioxidant responses were observed under combined FR+UV-A, particularly at a 50 µmol m^−2^ s^−1^, indicating that UV-A stimulated protective metabolic pathways associated with oxidative stress defense.

### Photochemical efficiency and gas exchange

Chlorophyll fluorescence parameters remained relatively stable across most treatments, indicating that the applied light regimes did not induce severe photoinhibition or irreversible damage to photosystem II. In basil, however, lower *F*_v_/*F*_m_ values under the combined FR+UV-A at 25 µmol m^−2^ s^−1^ suggest that low irradiance together with UV-A exposure increased excitation pressure and imposed mild physiological stress. This response corresponded with reductions in pigment concentration and lower PI, indicating reduced photochemical efficiency under these conditions. In contrast, the pronounced increase in PI under FR alone at 25 µmol m^−2^ s^−1^ intensity suggests that FR improved photosynthetic energy conversion and electron transport efficiency, potentially through enhanced canopy light interception and improved source–sink relationships (Fallah et al., 2024). Similar improvements in photochemical performance under FR-enriched lighting have been reported in leafy crops (Meng et al., 2024; Zhen and Bugbee, 2020).

In basil, the highest g_s_ values were observed under combined FR+UV-A at 50 µmol m^−2^ s^−1^, indicating enhanced stomatal opening and potentially greater transpirational and gas-exchange activity. UV-A has been reported to stimulate stomatal responses via blue/UV-A photoreceptor-mediated signaling pathways, leading to increased stomatal aperture and improved CO_2_ diffusion (Hernández and Kubota, 2020). In parsley, g_s_ declined under 25 µmol m^−2^ s^−1^, suggesting stronger stomatal regulation under reduced irradiance. High-intensity treatments maintained comparatively greater stomatal conductance, particularly under FR-enriched spectra, indicating that sufficient photon flux was required to sustain active gas exchange and photosynthetic activity.

### Amino acid and nitrate accumulation

Total amino acid and nitrate accumulation were markedly influenced by light intensity and spectral composition. In both basil and parsley, amino acid concentration generally increased under RBW at 100 µmol m^−2^ s^−1^, suggesting that greater photosynthetic photon flux enhanced the availability of carbon skeletons required for nitrogen assimilation and amino acid biosynthesis through the GS–GOGAT pathway (Liu et al., 2025). Adequate irradiance likely improved carbon fixation and energy supply, thereby supporting nitrogen metabolism and amino acid production (Liu and van Iersel, 2023; Meng et al., 2024).

In basil, amino acid concentration remained relatively high under FRat 100 µmol m^−2^ s^−1^, although slight reductions compared with the RBW suggest that part of the assimilated carbon may have been redirected toward rapid shoot growth and secondary metabolite production under FR. Similar responses have been reported in FR-treated crops where enhanced canopy expansion and biomass accumulation increased sink demand for photoassimilates, thereby modifying carbon partitioning patterns (Carotti et al., 2024). Under 50 µmol m^−2^ s^−1^ and 25 µmol m^−2^ s^−1^, amino acid accumulation declined substantially, particularly under FR+UV-A treatments, indicating that limited availability of photosynthetic carbon constrained nitrogen assimilation under reduced irradiance.

In parsley, FR at high intensity reduced amino acid content relative to the RBW, suggesting that FR-induced changes in growth and assimilate allocation influenced nitrogen metabolism. However, the addition of UV-A partially restored amino acid accumulation under FR-enriched conditions, indicating that UV-A may stimulate stress-responsive nitrogen metabolism and the synthesis of amino acid-related protective compounds. UV-A-mediated enhancement of stress-related metabolites and osmoprotective amino acids has previously been associated with activation of antioxidant and defense pathways under modified light environments Morales et al., 2010).

In parsley, nitrate concentration increased markedly under high-intensity FR and FR+UV-A treatments, suggesting that nitrate uptake exceeded nitrate reduction under these spectral conditions. This imbalance may indicate that nitrate reductase activity did not fully meet the demand for nitrate assimilation, resulting in nitrate accumulation within plant tissues (Santamaria, 2006; Signore et al., 2020). In contrast, lower nitrate levels observed under 25 µmol m^−2^ s^−1^ and some FR+UV-A treatments may reflect enhanced nitrate assimilation relative to uptake or reduced nitrate transport associated with slower growth and altered redox metabolism under limited light availability.

### PCA-based integration of physiological responses

The PCA analysis revealed clear treatment-associated separation patterns among physiological, biochemical, and growth-related traits, indicating that basil and parsley differed in their metabolic and physiological acclimation to spectral composition and light intensity. In parsley, differences were more closely associated with amino acids, nitrate, TPC, and TFC, particularly under FR+UV-A treatments. This clustering pattern suggests that spectral enrichment promoted stronger regulation of nitrogen metabolism and phenylpropanoid biosynthesis. UV-A radiation activates UVR8–HY5 signaling pathways, which transcriptionally regulate flavonoid and phenolic biosynthetic genes and stimulate antioxidant metabolism (Morales et al., 2010). The simultaneous association of nitrate with phenolic and flavonoid traits further suggests that nitrogen accumulation and secondary metabolism were closely linked under these spectral conditions.

In contrast, basil was more strongly associated with chlorophylls, carotenoids, *F*_v_/*F*_m_, PI, and stomatal conductance, indicating that spectral treatments primarily influenced photosynthetic performance and light-harvesting efficiency. FR likely altered phytochrome photoequilibrium, resulting in enhanced chlorophyll accumulation, increased photochemical activity, and improved physiological performance in FR-enriched environments (Zhen and Bugbee, 2020; Meng et al., 2024).

The PCA also demonstrated that FR and UV-A induced distinct metabolic adjustments that varied by species and light intensity. FR-enriched treatments were generally associated with enhanced photosynthetic and growth-related parameters, whereas UV-A was more closely linked with antioxidant and secondary metabolite accumulation. Under lower PPFD, UV-A appeared to impose greater physiological constraints in basil, while simultaneously stimulating defense-related metabolic pathways in parsley. These multivariate relationships highlight the importance of species-specific spectral management in controlled-environment agriculture and demonstrate that interactions among FR, UV-A, and light intensity regulate distinct aspects of plant acclimation, including growth, photosynthesis, and antioxidant responses.

## Conclusion

This study demonstrated that the effects of FR differed markedly when applied alone versus in combination with UV-A, and that responses varied with light intensity and crop species. FR alone generally promoted vegetative growth, chlorophyll accumulation, photosynthetic performance, and biomass production, particularly under 50- 100 µmol m^−2^ s^−1^ light. In basil, FR-enriched treatments strongly enhanced growth and photosynthetic efficiency, indicating improved light harvesting and carbon assimilation. In contrast, the addition of UV-A together with FR shifted plant responses toward secondary metabolism and antioxidant accumulation. Under several conditions, particularly in basil under high irradiance, UV-A reduced biomass accumulation, pigment content, and photochemical performance compared with FR alone, indicating a partially antagonistic interaction between UV-A and FR for growth-related traits. However, FR+UV-A treatments increased levels of phenolics, flavonoids, anthocyanins, antioxidant activity, and nitrogen-related metabolites, especially in parsley, suggesting a stronger role of UV-A in stress-related metabolic acclimation and photoprotection.

PCA analysis further confirmed distinct treatment-associated acclimation patterns, where basil was more strongly associated with pigments, fluorescence parameters, stomatal conductance, and amino acid accumulation, whereas parsley was more closely related to nitrate, phenolics, flavonoids, and antioxidant activity. Overall, FR primarily enhanced productivity-related traits, whereas FR+UV-A promoted quality-related metabolic responses. These findings demonstrate that UV-A may either complement or partially counteract FR effects, depending on the irradiance level and the physiological target, highlighting the importance of species-specific spectral optimization in controlled-environment agriculture.

Although the results suggest that adding UV-A to the FR treatment altered plant growth and secondary metabolite accumulation compared with FR alone, these responses should not be interpreted as conclusive evidence of synergistic or antagonistic interactions. Because an RBW + UV-A treatment was not included. The independent contribution of UV-A could not be separated from the combined FR + UV-A treatment. Future studies should include an RBW + UV-A treatment to better distinguish the individual and combined effects of FR and UV-A light.

## Data Availability

The original contributions presented in the study are included in the article/**Supplementary Material**, further inquiries can be directed to the corresponding author.
