# Supplementary material for: Interactive effects of FR and UV-A on growth and phytochemical content of basil and parsley grown in an indoor vertical farm
Source: Front Plant Sci. 2026 Jul 22;17:1843306. doi: 10.3389/fpls.2026.1843306 (PMC13438295; doi:10.3389/fpls.2026.1843306)
Supplement: Supplementary file 1 [file Table1.docx]

Interactive Effects of Spectrum and Intensity of RBW, FR and UV-A on Growth, Photochemical Performance, and Antioxidant Properties of Basil and Parsley Grown in Indoor Vertical Farm

Md Obyedul Kalam Azad and Most Tahera Naznin *

Department of Agriculture, Veterinary and Rangeland Sciences, University of Nevada, Reno, UNR Extension and Agricultural Experimental Station, Las Vegas, NV, 89030

*****	Correspondence: M.T. Naznin: Email: [mnaznin@unr.edu](mailto:mnaznin@unr.edu)

**Table S1.** Two-way ANOVA showing the effects of light intensity (I), light spectrum (S), and their interaction (I × S) on plant growth parameters of basil.

| Source of variation | Plant height | Leaf No | Leaf area | Root length | Fresh weight | Dry weight |
| --- | --- | --- | --- | --- | --- | --- |
| Intensity (I) | *** | *** | *** | ** | *** | *** |
| Spectrum (S) | *** | *** | *** | ns | *** | *** |
| I × S | *** | *** | *** | * | *** | *** |

**Significance levels:** *** p < 0.001 ; ** p < 0.01 ; p < 0.05 ; ns = not significant

**Table S2.** Two-way ANOVA showing the effects of light intensity (I), light spectrum (S), and their interaction (I × S) on plant growth parameters of parsley.

| Source of variation | Plant height | Leaf No | Leaf area | Root length | Fresh weight | Dry weight |
| --- | --- | --- | --- | --- | --- | --- |
| Intensity (I) | *** | ns | *** | ** | *** | *** |
| Spectrum (S) | *** | ns | *** | * | *** | *** |
| I × S | *** | ns | *** | * | *** | *** |

**Significance levels:** *** p < 0.001 ; ** p < 0.01 ; p < 0.05 ; ns = not significant

**Table S3.** Two-way ANOVA showing the effects of light intensity (I), light spectrum (S), and their interaction (I × S) on TPC and TFC of basil.

| Source of variation | TPC (Basil) | TPC (Parsley) | TFC (Basil) | TFC (Parsley) |
| --- | --- | --- | --- | --- |
| Intensity (I) | *** | *** | *** | *** |
| Spectrum (S) | *** | *** | *** | *** |
| I × S interaction | *** | *** | *** | *** |

**Significance levels:** *** p < 0.001 ; ** p < 0.01 ; p < 0.05 ; ns = not significant

Table S4. Two-way ANOVA showing the effects of light intensity (I), light spectrum (S), and their interaction (I × S) on pigment content (Chl *a*, Chl *b*, and carotenoids) in basil and parsley.

| **Trait** | **Source of variation** | **Basil** | **Parsley** |
| --- | --- | --- | --- |
| **Chl *a*** | Intensity (I) | *** | *** |
|  | Spectrum (S) | *** | *** |
|  | I × S | *** | *** |
| **Chl *b*** | Intensity (I) | *** | *** |
|  | Spectrum (S) | *** | *** |
|  | I × S | *** | *** |
| **Car** | Intensity (I) | *** | *** |
|  | Spectrum (S) | *** | *** |
|  | I × S | *** | *** |

**Significance levels:** *** p < 0.001 ; ** p < 0.01 ; p < 0.05 ; ns = not significant

**Table S5.** Two-way ANOVA showing the effects of light intensity (I), light spectrum (S), and their interaction (I × S) on chlorophyll fluorescence (*F*_v_/*F*_m_), performance index (PI), and stomatal conductance (g_s_) in basil and parsley.

| **Trait** | **Source of variation** | **Basil** | **Parsley** |
| --- | --- | --- | --- |
| *F*_v_/*F*_m_ | Intensity (I) | *** | *** |
|  | Spectrum (S) | *** | *** |
|  | I × S | *** | *** |
| **PI** | Intensity (I) | *** | *** |
|  | Spectrum (S) | *** | *** |
|  | I × S | *** | *** |
| **g_s_** | Intensity (I) | *** | *** |
|  | Spectrum (S) | *** | *** |
|  | I × S | *** | *** |

*** p < 0.001, ** p < 0.01, * p < 0.05, ns = not significant

**Table S6.** Two-way ANOVA showing the effects of light intensity (I), light spectrum (S), and their interaction (I × S) on total amino acid and nitrate contents in basil and parsley.

| **Trait** | **Source of variation** | **Basil** | **Parsley** |
| --- | --- | --- | --- |
| **Total amino acids** | Intensity (I) | *** | *** |
|  | Spectrum (S) | *** | *** |
|  | I × S | *** | *** |
| **Total nitrate** | Intensity (I) | *** | *** |
|  | Spectrum (S) | *** | *** |
|  | I × S | *** | *** |

*** p < 0.001, ** p < 0.01, * p < 0.05, ns = not significant
